# The FlgT Protein Is Involved in *Aeromonas hydrophila* Polar Flagella Stability and Not Affects Anchorage of Lateral Flagella

**DOI:** 10.3389/fmicb.2016.01150

**Published:** 2016-07-26

**Authors:** Susana Merino, Juan M. Tomás

**Affiliations:** Departamento de Genética, Microbiología y Estadística, Sección Microbiologia, Virología y Biotecnología, Facultad de Biología, Universidad de BarcelonaBarcelona, Spain

**Keywords:** *Aeromonas*, *flgT*, polar and lateral flagella

## Abstract

*Aeromonas hydrophila* sodium-driven polar flagellum has a complex stator-motor. Consist of two sets of redundant and non-exchangeable proteins (PomA/PomB and PomA_2_/PomB_2_), which are homologs to other sodium-conducting polar flagellum stator motors; and also two essential proteins (MotX and MotY), that they interact with one of those two redundant pairs of proteins and form the T-ring. In this work, we described an essential protein for polar flagellum stability and rotation which is orthologs to *Vibrio* spp. FlgT and it is encoded outside of the *A. hydrophila* polar flagellum regions. The *flgT* was present in all mesophilic *Aeromonas* strains tested and also in the non-motile *Aeromonas salmonicida*. The *A. hydrophila* Δ*flgT* mutant is able to assemble the polar flagellum but is more unstable and released into the culture supernatant from the cell upon completion assembly. Presence of FlgT in purified polar hook-basal bodies (HBB) of wild-type strain was confirmed by Western blotting and electron microscopy observations showed an outer ring of the T-ring (H-ring) which is not present in the Δ*flgT* mutant. Anchoring and motility of proton-driven lateral flagella was not affected in the Δ*flgT* mutant and specific antibodies did not detect FlgT in purified lateral HBB of wild type strain.

## Introduction

Motility represents an important advantage for bacteria in moving toward favouable conditions, in avoiding of detrimental environments, or in having successful competes with other microorganisms (Frenchel, 2002). The motility organ used by many bacteria to move through liquid or semisolid media is the flagellum, although their number and placement shows differences between species. Flagella are supramolecular reversible rotary complexes anchored in the bacterial surface and made up of many different proteins. A flagellum consists of a filament, a hook and a basal body. The basal body is embedded in the cell envelope and works as a reversible rotary motor, whereas the hook and the filament function as a universal joint and a propeller, respectively (Berg, 2003; Macnab, 2003). The flagella basal body consists in some rings that allow the flagellum rod crossing through the cell envelope, a reversible rotary motor and a protein export apparatus that translocate the flagellar components. In Gam-negative bacteria there are three rings involved: L-, P-, and MS-rings. The L-ring is composed of the FlgH protein and outer membrane-embedded. The P-ring is composed of the FlgI protein, lies in the periplasmic space and is associated with the peptidoglycan layer. Both rings form the LP ring complex that functions as a molecular bushing. The MS-ring is composed of the FliF protein and inner membrane-embedded, being the starting point for motor assembly (Ueno et al., 1992; Macnab, 1996; DeRosier, 1998). The flagellum motor is made of a rotor and about a dozen stator complexes. The rotor is composed of an axial rod and the C-ring, which assemble around the MS-ring and the export apparatus. The C-ring lies in the cytoplasm, is composed of the FliM, FliN, and FliG proteins and is the site of torque generation and switching the direction of flagellum rotation (Khan et al., 1991; Francis et al., 1994; Katayama et al., 1996). Above the C-ring, surrounding the MS-ring in the inner membrane and attached to the peptidoglycan layer are the stators complex. Each stator complex is made up of two membrane proteins with an apparent 4:2 stoichiometry. These membrane proteins constitute an ion channel that transform the flow of proton or sodium ions across the cytoplasmic membrane into the energy required for flagella motor rotation (McCarter, 2001; Yorimitsu and Homma, 2001; Blair, 2003; Terashima et al., 2008). Most bacterial flagella use a single type of stator complex: proton- or sodium-dependent. The proton-dependent stator complex is made up of MotA and MotB, like in *Escherichia coli* and *Salmonella enterica* serovar Typhimurium flagella (Blair and Berg, 1990; Stolz and Berg, 1991; Macnab, 1996). The sodium-dependent stator complex is made up of PomA and PomB, as in *Vibrio* species (Asai et al., 1997; McCarter, 2001; Yorimitsu and Homma, 2001) or MotP and MotS, as in alkaliphilic *Bacillus* species (Ito et al., 2004). However, the flagella motor of some bacterial species is energized by two different sets of stator complexes. In *Bacillus subtilis*, MotAB, and MotPS; and in *Shewanella oneidensis* MR-1, MotAB, and PomAB, supports flagellar rotation by proton and sodium ions flow, respectively (Ito et al., 2004; Paulick et al., 2009). Nevertheless, in *Aeromonas hydrophila*, PomAB, and PomA_2_B_2_ are both sodium-coupled stator complexes with different sensitivity to sodium concentrations (Wilhelms et al., 2009) and in *Pseudomonas aeruginosa* PAO1, MotAB, and MotCD are both proton-dependent stator complex (Doyle et al., 2004; Toutain et al., 2005). Surrounding the conserved stator structure, different bacterial species display various additional components. The lateral flagella proton-dependent stator of *Vibrio parahaemolyticus* requires an additional protein, MotY, with a peptidoglycan-binding domain (Stewart and McCarter, 2003). The polar flagellum sodium-dependent stator of *Vibrio* species, *S. oneidensis* MR-1 and *A. hydrophila* contain two additional proteins: MotX and MotY, which make up a beneath structure of P-ring which is named T-ring (Okabe et al., 2002; Yagasaki et al., 2006; Terashima et al., 2008; Koerdt et al., 2009). Furthermore, surrounding the polar-flagellum LP-rings of *Vibrio* species is the H-ring, which is composed of FlgT protein. The T- and H-rings are required for properly assembly of the PomAB stator complex around the rotor in *Vibrio* species (Terashima et al., 2006, 2010, 2013).

*Aeromonas* are found ubiquitously in the environment, but are mainly associated with fresh or estuarine water. They are the causative agent of wide spectrum of diseases in man and animals and some species are becoming food and waterborne pathogens of increasing importance (von Graevenitz, 2007; Ghenghesh et al., 2008). Mesophilic *Aeromonas* have a single polar flagellum produced constitutively and 50–60% of clinical isolates also have lateral inducible flagella. Fully functional polar and lateral flagella are essential for a proper attachment, biofilms formation, and colonization (Merino et al., 1997; Rabaan et al., 2001; Gavín et al., 2002). Although, both flagella types are structurally similar, they have some differences at the export apparatus and the motor. The FliO protein is only present in the polar flagella export apparatus. The lateral flagella are proton-driven and their stator complex made up of two proteins, LafT and LafU (Canals et al., 2006a; Molero et al., 2011). However, the polar flagellum is sodium-driven and their stator complex consists of two sets of membrane proteins: PomAB and PomA_2_B_2_ (Wilhelms et al., 2009), as well as two essential proteins: MotXY, which make up the T-ring (Molero et al., 2011).

In this study, we reported a protein orthologous to FlgT of *Vibrio* spp., which present in all mesophilic *Aeromonas* and is encoded outside of the polar flagellum regions, which is involved in the stability and rotation of an unsheathed flagellum sodium-driven with two different stator complex.

## Materials and Methods

### Bacterial Strains, Plasmids, and Growth Conditions

Bacterial strains and plasmids used in this study are listed in **Table 1**. *E. coli* strains were grown on Luria-Bertani (LB) Miller broth and LB Miller agar at 37°C. *Aeromonas* strains were grown either in tryptical soy broth (TSB) or agar (TSA) at 25°C. When required ampicillin (100 μg/ml), kanamycin (50 μg/ml), tetracycline (20 μg/ml), chloramphenicol (25 μg/ml), rifampicin (100 μg/ml), and spectinomycin (50 μg/ml) were added to the different media. Media were supplemented with 0.2% (w/v) L-arabinose to induce recombinant proteins expression under the arabinose promoter on pBAD33.

**Table 1:** Bacterial strains and plasmid used in this study.

Strain or plasmid	Genotype and/or phenotype^a^	Reference
**Strains**		
*Aeromonas hydrophila*		
AH-3	*A. hydrophila* wild type, serogroup O :34	Merino et al., 1991
ATCC7966^T^	*A. hydrophila* wild type	Seshadri et al., 2006
AH-405	AH-3, spontaneous Rif^r^	Altarriba et al., 2003
ATCC7966-Rif	ATCC7966^T^, spontaneous Rif^r^	This work
AH-3Δ*flgT*	AH-405; Δ*flgT*	This work
ATCCΔAHA1089	ATCC7966-Rif; ΔAHA_1089	This work
AH-3::*flaA*Δ*flaB*	AH-405; *flaA*::Km^r^; Δ*flaB*	Canals et al., 2006b
AH-3:: *flhA*	AH-405; *flhA*::Km^r^	Canals et al., 2006b
AH-3Δ*lafA*	AH-405; Δ*lafA*	Wilhelms et al., 2013
AH-3::*flaA*Δ*flaBflgT*	AH-3::*flaA*::Km^r^; Δ*flaB*; Δ*flgT*	This work
AH-3Δ*lafAflgT*	AH-3Δ*lafA*; Δ*flgT*	This work
AH-3::*flrA*	AH-405; *flrA*::Km^r^	Wilhelms et al., 2011
AH-3::*flrBC*	AH-405; *flrB*::pSF, Km^r^	Wilhelms et al., 2011
AH-3::*fliA*_P_	AH-405; *fliA*_P_:: Km^r^	Canals et al., 2006b
AH-3::*lafK*	AH-405; *lafK*::Km^r^	Canals et al., 2006a
AH-3::*lafS*	AH-405; *lafS*::Km^r^	Wilhelms et al., 2013
***Escherichia coli***		
DH5α	F^-^ *endA hdsR17*(rk^-^ mk^+^) *supE44 thi-1 recA1 gyr-A96*ϕ80*lacZ*	Hanahan, 1983
MC1061λpir	*thi thr1 leu6 proA2 his4 argE2 lacY1 galK2 ara14 xyl5 supE44*λ *pir*	Rubires et al., 1997
**Plasmids**		
pLA2917	Cosmid vector, Tc^r^ Km^r^	Allen and Hanson, 1985
pLA-FLGT	pLA2917 with AH-3 *flgT*, Tc^r^.	This work
pRK2073	Helper plasmid, Sp^r^	Rubires et al., 1997
pGEMT	Cloning vector, Ap^r^.	Promega
pDM4	Suicide plasmid, *pir* dependent with *sacAB* genes, oriR6K, Cm^r^.	Milton et al., 1996
pDM-AHA1089	pDM4ΔAHA_1089 of ATCC7966^T^, Cm^r^.	This work
pDM-FLGT	pDM4Δ*flgT* of AH-3, Cm^r^.	This work
pET-30 Xa/LIC	IPTG inducible expression vector Km^R^	Novagen
pET-30-FlgT	pET-30 Xa/LIC with *A. hydrophila* AH-3 *flgT*	This study
pBAD33	pBAD33 arabinose-induced expression vector with Cm^r^	Guzman et al., 1995
pBAD33-FLGT	pBAD33 with AH-3 *flgT* gen, Cm^r^	This work

*^a^ Km^r^, kanamycin resistant; Ap^r^, ampicillin resistant; Rif^r^, rifampicin resistant; Cm^r^, chloramphenicol resistant; Sp^r^, spectinomycin resistant; Tc^r^, tetracycline resistant.*

### Motility Assays (Swarming and Swimming)

Fresh bacterial grown colonies were transferred with a sterile toothpick onto the center of a soft agar plate (1% tryptone, 0.5% NaCl, 0.25% agar). Plates were incubated face up for 24–48 h. at 25°C and motility was assessed by examining the migration of bacteria through the agar from the center toward the periphery of the plate. Moreover, swimming motility was assessed by light microscopy observations in liquid media.

### Transmission Electron Microscopy (TEM)

Bacterial suspensions were placed on Formvar-coated grids and negative stained with a 2% solution of uranyl acetate pH 4.1. Preparations were observed on a Jeol JEM 1010 transmission electron microscope.

### DNA Techniques

DNA manipulations were carried out according to standard procedures (Sambrook et al., 1989). DNA restriction endonucleases were obtained from Promega. T4 DNA ligase and alkaline phosphatise were obtained from Invitrogen and GE Healthcare, respectively. PCR was performed using the BioTaq DNA polymerase (Ecogen) in a Gene Amplifier PCR System 2400 Perkin Elmer Thermal Cycler. Colony hybridizations were carried out by colony transfer onto positive nylon membranes (Roche) and then lysed according to the manufacturer’s instructions. Probe labeling with digoxigenin, hybridization and detection (GE Healthcare) were carried out as recommended by the suppliers.

### Nucleotide Sequencing and Computer Sequence Analysis

Plasmid DNA for sequencing was isolated by Qiagen plasmid purification kit (Qiagen, Inc. Ltd.) as recommended by the suppliers. Double-strand DNA sequencing was performed by using the Sanger dideoxy-chain termination method (Sanger et al., 1977) with the BigDye Terminator v3.1 cycle sequencing kit (Applied Biosystem). Custom-designed primers used for DNA sequencing were purchased from Sigma–Aldrich.

DNA sequence was translated in all six frames, and their deduced amino acid sequences were inspected in the GenBank, EMBL, and SwissProt databases by using the BLASTX, BLASTP, or PSI-BLAST network service at the National Center for Biotechnology Information (NCBI) (Altschul et al., 1997). Protein family profile was performed using the Protein Family Database Pfam at the Sanger Center (Bateman et al., 2002).

### RT-PCR

Total RNA was isolated from *A. hydrophila* AH-3, AH-3::*flrA*, AH-3::*flrBC*, AH-3::*fliA*_p_, AH-3::*lafK*, and AH-3::*lafS* which were grown at 25°C in liquid media (TSB) or plates (TSA) by RNA Protect Bacteria Reagent (Qiagen) and RNeasy Mini kit (Qiagen). To ensure that RNA was devoid of contaminating DNA, the preparation was treated with RNase-free TurboDNase I (Ambion). First-strand cDNA synthesis was carried out using the Thermoscript RT-PCR system (Invitrogen) and random primers on 5 μg of total RNA DNase-digested, according to the manufacturer’s instructions. PCR without reverse transcriptase was also performed to confirm the absence of contaminating DNA in the RNA sample. The second strand synthesis and subsequent DNA amplification of *flgT* internal fragment was carried out using the BioTaqDNA polymerase (Bioline) and the pair of oligonucleotides5′-CAGTGGCTGG ACGAGAAC-3′ and 5′- TTCCAATACTGCCAGATCC-3′ designed using the Prime program from the Genetics Computer Group package (Madison, Wisconsin). Amplicons were visualized by agarose gel electrophoresis with ethidium bromide staining. *A. hydrophila* ribosomal 16S primers were used as a control of cDNA template.

### Constructions of Defined Mutants

The single defined insertion ATCCΔAHA1089 and AH-3Δ*flgT* were obtained by allelic exchange as described by Milton et al. (1996). Briefly, DNA regions upstream and downstream of AHA_1089 of *A. hydrophila* ATCC7966^T^ were ampli-fied using the primer pairs A1 (5′-CGC*GGATCCAAT*CTTGACCACCACCACT-3′) and B1 (5′-CCCATCCACTAAACT TAAACAGGCGTAGACCTCGTCTGT-3′), and C1 (5′-TGTTTAAGTTTAGTGGAT GGGGATCAGTTCCGCATCCAG-3′) and D1 (5′-CGC*GGATCC*CTCGATGGTCCA ATCCAT-3′) in two sets of asymmetric PCRs to amplify DNA fragments of 610 (A1B1) and 674 (C1D1) bp, respectively. Regions upstream and downstream of *flgT* of *A. hydrophila* AH-3 were amplified using the primer pairs A1 and B2 (5′-CCCATCCA CTAAACTTAAACACTGTTCACGGGCATAGAC-3′), and C2 (5′-TGTTTAAGTTT AGTGGATGGGGTGATAGGCCAGAACGAAC-3′) and D2 (5′- CGC*GGATCC*TGT CAGCTGTTTGGTTACG-3′) in two sets of asymmetric PCRs to amplify DNA fragments of 617 (A1B2) and 619 (C2D2) bp, respectively. DNA fragment A1B1 and C1D1 or A1B2 and C2D2 were annealed at their overlapping regions (underlined letters in primers B and C) and amplified as a single fragment using primers A1 and D1 or A1 and D2. The AD fusion products were purified, *Bam*HI digested (the *Bam*HI site is double-underlined in primer A1, D1, and D2), ligated into *BglII*-digested and phosphatase-treated pDM4 vector (Milton et al., 1996) and electroporated into *E. coli* MC1061*(λpir*) and plated on chloramphenicol plates at 30°C to obtain pDM-AHA1089 and pDM-FLGT plasmids, respectively. Introduction of the plasmids into *A. hydrophila* ATCC7966-Rif or AH-405 rifampicin-resistant (Rif^r^), was performed by triparental matings using the *E. coli* MC1061 *(λpir*) containing the insertion constructs and the mobilizing strain HB101/pRK2073. Transconjugants were selected on plates containing chloramphenicol and rifampicin. PCR analysis confirmed that the vector had integrated correctly into the chromosomal DNA. After sucrose treatment, transformants that were rifampicin-resistant (Rif^r^) and chloramphenicol sensitive (Cm^S^) were chosen and confirmed by PCR.

The mutants AH-3::*flaA*Δ*flaBflgT* and AH-3Δ*lafA*Δ*flgT* were obtained by introduction of the pDM-FLGT plasmid into *A. hydrophila* AH-3::*flaA*Δ*flaB* and AH-3::*lafA*, respectively, by triparental mating using the *E. coli* MC1061 *(λpir*) containing the plasmid and the mobilizing strain HB101/pRK2073. Transconjugants were selected on plates containing chloramphenicol, kanamycin and rifampicin or chloramphenicol and rifampicin, respectively. After sucrose treatment, transformants that were rifampicin and kamycine-resistant or rifampicin-resistent and chloramphenicol sensitive, respectively, were chosen, and confirmed by PCR.

### Plasmid Constructions

Plasmid pBAD33-FLGT containing the complete *flgT* gene from *A. hydrophila* AH-3 under the arabinose promoter (p_BAD_) on pBAD33 (Guzman et al., 1995) was obtained by PCR amplification of genomic DNA. Oligonucleotides 5′-TCTAGACACGGTTCTGTGGTCTGTA-3′ and 5′-GTCGACGG GACCGCTCTATCCTACT-3′ generated a band of 1319bp containing the *flgT* gene (the *Xba*I site is underlined and the *Sal*I site double-underlined). The amplified band containing the *flgT* gen was ligated into pGEM-Teasy (Promega) and transformed into *E. coli* XL1-Blue. The DNA insert was recovered by *Xba*I and *Sal*I restriction digestion and ligated into *Xba*I-*Sal*I digested pBAD33 vector to construct the pBAD33-FLGT plasmid. Recombinant plasmid was introduced by electroporation into the *E. coli* DH5α (Hanahan, 1983) and was sequenced. For complementation assay, the recombinant plasmid was introduced into the AH-3Δ*flgT* mutant (Rif^r^) by triparental mating using the *E. coli* DH5α containing the pBAD33-FLGT plasmid and the mobilizing strain HB101/pRK2073. Transconjugants were selected on plates containing chloramphenicol and rifampicin.

### Isolation of the *A. hydrophila* Polar Flagellar Hook-basal Bodies

Isolation of the *A. hydrophila* polar flagella HBBs was carried out from an overnight culture in T.S.B. (1000 ml) at 25°C as described by Terashima et al. (2006). Briefly, after cultivation, the cells were harvested in a sucrose solution (0.5 M sucrose, 50 mM Tris-HCl at pH 8.0) and converted into spheroplasts by adding lysozyme and EDTA to final concentrations of 0.1 mg/ml and 2 mM, respectively. After lysis of spheroplasts with 1% (w/v) Triton X-100, 5 mM MgSO4, and 0.1 mg/ml DNase I were added to reduce viscosity and then, 5 mM EDTA was added. Unlysed cells and cellular debris were recovered by centrifugation at 17.000 × *g* for 20 min. Polyethylene glycol 6000 and NaCl were added to the lysate to final concentrations of 2% and 100 mM, respectively, and flagella were collected by centrifugation at 27000 *g* for 30 min. The pellet was suspended in TET buffer [10 mM Tris-HCl at pH 8.0, 5 mM EDTA, 0.1% (w/v) Triton X-100]. To remove cellular debris, the suspension was centrifuged at 1.000 × *g* for 15 min at 4°C and the supernatant was centrifuged at 100.000 × *g* for 30 min. To dissociate the flagella into monomeric flagellin, the pellet was suspended in TET buffer and diluted 30-fold in 50 mM glycine-HCl (pH 3.5) containing 0.1% (w/v) Triton X-100 and shaken for 60 min at room temperature. After treatment, the mixture was centrifuged at 1.000 × *g* for 15 min at 4°C and supernatant centrifuged 150.000 × *g* for 40 min and pellet suspended in TET buffer.

### Anti-FlgT Polyclonal Serum

To obtain the *A. hydrophila* AH-3 FlgT we overexpressed *A. hydrophila* AH-3 *flgT* in *E. coli* using pET-30 Xa/LIC vector (Novagen). The *A. hydrophila* AH-3 *flgT* was amplified from AH-3 genomic DNA using primers PETflgTfor 5′-GGTATTGAGGGTCGCATGAAATTACCGCTGCTG-3′ and PETflgTrev 5′-AGAGGAGAGTTAGAGCCGCGGGCATTATACAAGAAG-3′. The PCR product was ligated into pET-30 Xa/LIC (Novagen) by their overlapping regions (underlined letters in primers) and electroporated into *E. coli* BL21(*λ*DE3). The His_6_-FlgT protein was overexpressed and cell lysates obtained as previously reported for other proteins (Canals et al., 2007; Jiménez et al., 2009). The total membrane fraction was obtained by ultracentrifugation (200.000 × *g* 30 min at 10°C), the His_6_-FlgT protein was solubilized and purified with a Ni^2+^-NTA agarose (Quiagen) as previously reported (Al-Dabbagh et al., 2008). Approximately 200 μg of purified AH-3 FlgT was emulsified with 1 ml of Freunds complete adjuvant and inoculated subcutaneously into adult New Zealand rabbits. Booster injections of the flagellin protein were administered 4 and 6 weeks later. Antibodies were obtained by bleeding 10 days after the booster injection.

### Immunological Methods

Western blot of whole cell proteins and supernatants from *Aeromonas* strains grown in T.S.B. at 25°C or purified polar and lateral flagella basal bodies, was performed as briefly described. Whole cells and supernatants came from equivalent numbers of cells harvested by centrifugation. The cell pellet was suspended in 50–200 μl of SDS PAGE loading buffer and boiled for 5 min.

After SDS-PAGE and transfer to nitrocellulose membrane at 1.3 A for 1 h, the membranes were blocked with bovine serum albumin (3 mg/ml), and probed with polyclonal rabbit anti-FlgT antibodies (1:1000). The unbound antibody was removed by three washes in PBS, and a goat anti-rabbit immunoglobulin G alkaline phosphatase conjugated secondary antibody (1:1000) was added. The unbound secondary antibody was removed by three washes in PBS. The bound conjugate was then detected by the addition of 5-bromo-4-chloroindolylphosphate disodium-nitroblue tetrazolium. Incubations were carried out for 1 h, and washing steps with 0.05% Tween 20 in phosphate-buffered saline were included after each incubation step.

### Adherence Assay to HEp-2 Cell

Adherence assay was conducted as a slight modification of that described by Carrello et al. (1988). Bacteria were grown statically in brain heart infusion broth (BHIB) at 25°C, harvested by gentle centrifugation (1,600 × *g*, 5 min), and resuspended in PBS (pH 7.2) at approximately 10^7^ CFU/ml (A_600_ = 0.07). The HEp-2 cell monolayer was infected with 1 ml of the bacterial suspension for 90 min at 37°C in 5% CO_2_. Following infection, the non-adherent bacteria were removed from the monolayer by three washes with PBS. The remaining adherent bacteria and the monolayers were then fixed in 100% methanol for 5 min. Methanol was removed by washing them with PBS, and the HEp-2 cells with the adherent bacteria were stained for 45 min in 10% (vol/vol) Giemsa stain (BDH) prepared in Giemsa buffer. The coverslips were air dried, mounted, and viewed by oil immersion under a light microscope. Twenty HEp-2 cells/coverslips were randomly chosen, and the number of bacteria adhering/HEp-2 cell was recorded. Assays were carried out in duplicates or triplicates.

### Biofilm Formation

Quantitative biofilm formation was performed in a microtiter plate as described previously (Pratt and Kotler, 1998), with minor modifications. Briefly, bacteria were grown on TSA and several colonies were gently resuspended in TSB (with or without the appropriated antibiotic); 100 μl aliquots were place in a microtiter plate (polystyrene) and incubated 48 h at 30°C without shaking. After the bacterial cultures were poured out, the plate was washed extensively with water, fixed with 2.5% glutaraldehyde, washed once with water and stained with 0.4% crystal violet solution. After solubilisation of the crystal violet with ethanol-acetone (80/20, v/v) the absorbance was determined at 570 nm.

## Results

### Identification of a New *Aeromonas* spp. Protein Essential for Motility

**FIGURE 1:**
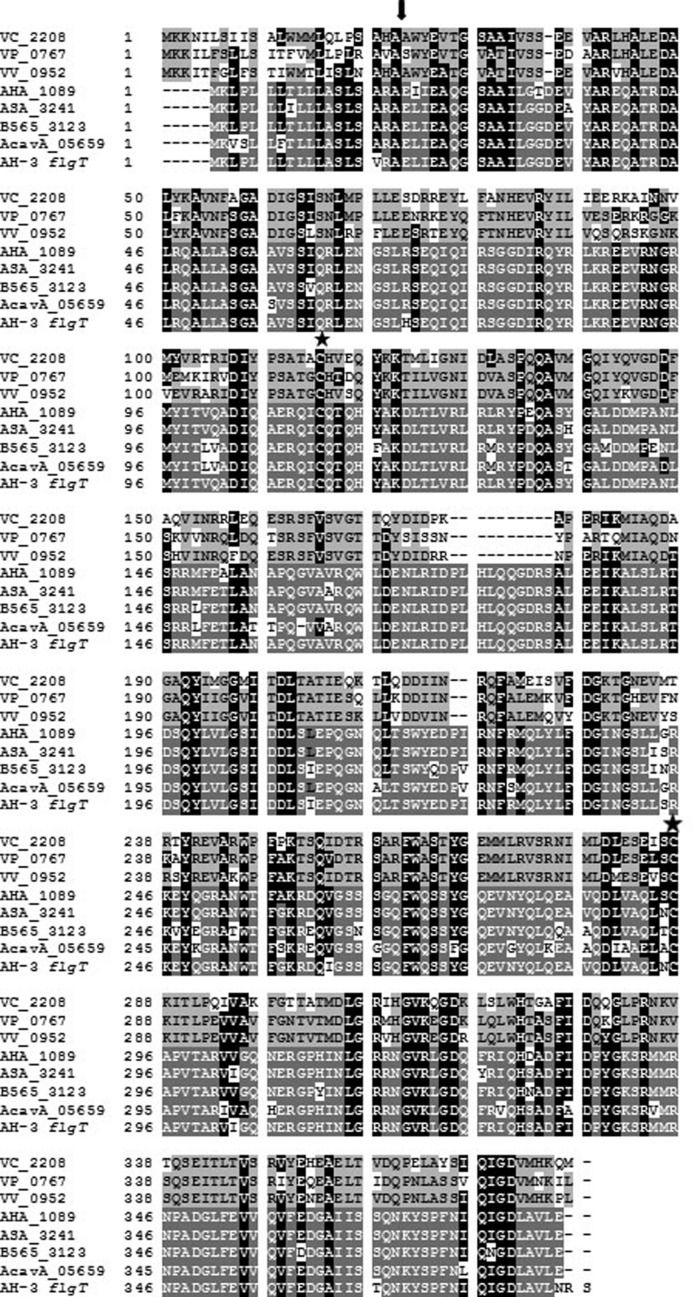
**Alignment of FlgT amino acid sequences of *Vibrio cholerae* (VC_2208), *Vibrio parahaemolyticus* (VP_0767), and *Vibrio vulnificus* FlgT (VV_0952); and the hypothetical protein of *Aeromonas hydrophila* ATCC7966^T^ (AHA_1089), *A. salmonicida* subsp. *salmonicida* A449 (ASA_3241), *A. veronii* B565 (B565_3123), *A. caviae* Ae398 (AcavA_05659), and *A. hydrophila* AH-3 (AH-3).** Black letters in light gray boxes indicate residues that matched in at least two of the three *Vibrio* sequences. White letters in dark gray boxes indicate residues that matched in at least three of the five *Aeromonas* sequences. White letters in black boxes indicate residues that are present in *Vibrio* and *Aeromonas* sequences. The arrowhead shows the site of signal sequence cleavage. The stars show the conserved cysteine residues.

Mesophilic *Aeromonas* have a constitutive unsheathed polar flagellum energized by sodium ions. The stator complex of *Aeromonas* polar flagellum is composed of two redundant pairs of membrane proteins: PomAB and PomA_2_B_2_, with different sensitivity to sodium concentrations; and two motility essential proteins (MotXY) which make up the T-ring (Wilhelms et al., 2009; Molero et al., 2011). In *Vibrio* spp. the sodium-driven polar flagellum shows a ring (H-ring) surrounding the LP-rings, which is composed of the FlgT protein and may be involved in the assembly of MotXY to the basal body (Cameron et al., 2008; Terashima et al., 2010). The analysis of *A. hydrophila* ATCC7966^T^, *A. salmonicida* subsp. *salmonicida* A449, *A. veronii* B565 and *A. caviae* Ae398 genome sequences (Seshadri et al., 2006; Reith et al., 2008; Beatson et al., 2011; Li et al., 2011) revealed an open reading frame (AHA_1089, ASA_3241, B565_3123, and AcavA_05659, respectively), annotated as hypothetical protein which deduced amino acid sequences exhibit 27–28% identity, 46–48% similarity and *E*-value of 1e-34 to 6e-36 to *Vibrio spp*. FlgT (**Figure 1**). The *A. hydrophila* AH-3 genomic library was screened by colony blotting using an AHA_1089 DNA probe leading to the identification of clone pLA-FLGT (Nogueras et al., 2000), which carries the entire *flgT* gene. *A. hydrophila* AH-3 FlgT is predicted to be 386 amino acids in length and exhibits 96% identity/98% similarity to *A. hydrophila* ATCC7966^T^ AHA_1089. Furthermore, *Aeromonas* FlgT harbor a signal peptide for secretion with a cleavage site between Ala^18^ and Glu^19^ (**Figure 1**), which suggest it is translocated to the periplasmic space like MotX and MotY. As described in *Vibrio* FlgT, the *Aeromonas* FlgT show two conserved cysteine residues that might form a disulfide bond for protein stabilization (**Figure 1**).

**FIGURE 2:**
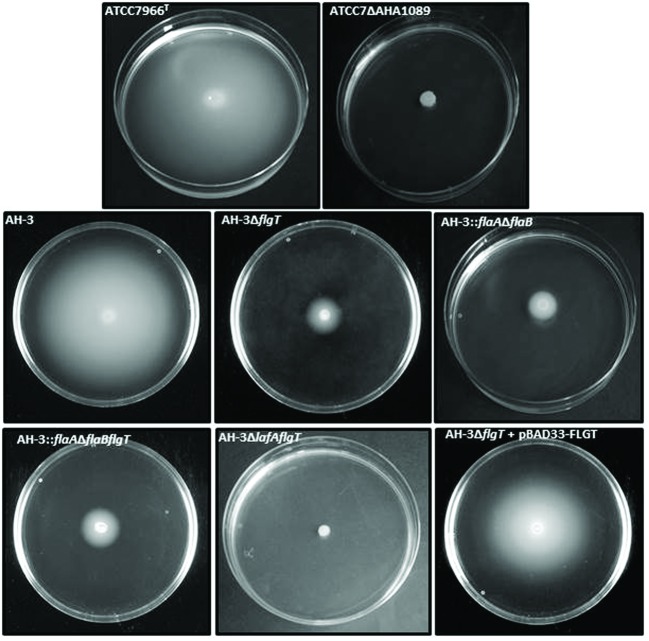
**Motility of *A. hydrophila* strains: AH-3, ATCC7966^T^, ATCCΔAHA1089, AH-3Δ*flgT*, AH-3::*flaA*Δ*flaB* (AH-3 non-polar flagellum mutant), AH-3::*flaA*Δ*flaBflgT*, AH-3Δ*lafA*Δ*flgT*, and AH-3Δ*flgT* harboring plasmid pBAD33-FLGT grown 20 h at 25°C on soft agar.** The mutant complemented with pBAD33 plasmid was grown with 0.2% L-arabinose.

To investigate the role of this protein in the *Aeromonas* motility, defined insertion mutants were created in two different *A. hydrophila* strains: ATCC7966^T^, which only possess polar flagellum (ATCCΔAHA1089), and AH-3, which possess constitutive polar flagellum and inducible lateral flagella (AH-3Δ*flgT*). Motility assays in liquid media by light microscopy showed that AHA_1089 and *flgT* mutations abolish swimming motility in ATCC7966^T^ and AH-3, respectively. However, whereas motility in soft agar was abolished in the ATCCΔAHA1089 mutant, in the AH-3Δ*flgT* mutant it causes a highly decrease of radial expansion (68% reduction), in relation to the wild-type. The radial expansion of AH-3Δ*flgT* mutant was similar to those observed in mutants without polar flagella as AH-3Δ*flaAB* mutant (Canals et al., 2006b) (**Figure 2**).

**FIGURE 3:**
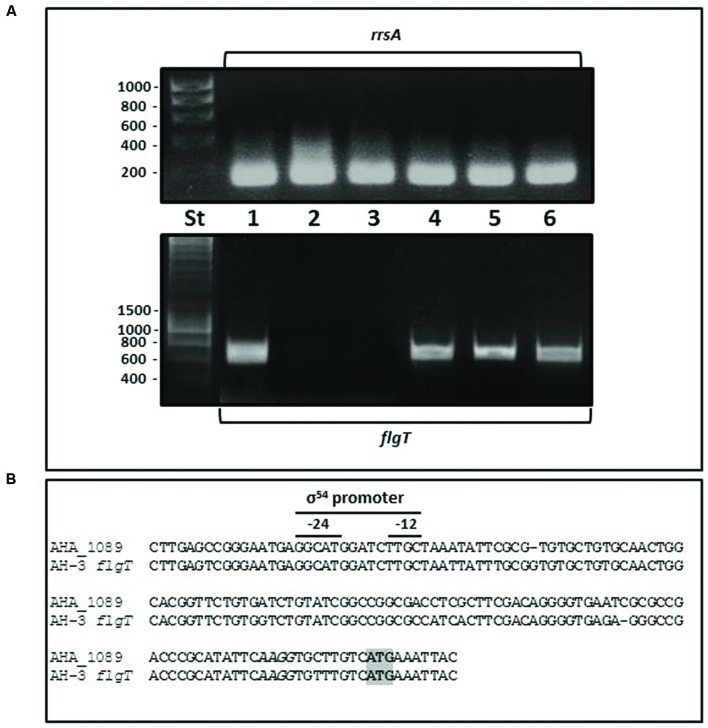
**(A)** RT-PCR amplification of *A. hydrophila flgT* internal fragments from cDNA of *A. hydrophila* AH-3 (1), AH3::*flrA* (2), AH-3::*flrBC* (3), AH-3::*fliA*_P_ (4), AH-3::*lafK* (5), and AH-3::*lafS* (6) mutants. DNA molecular marker (St). *A. hydrophila* ribosomal 16S (*rrsA*) amplification was used as a control for cDNA template. RT-PCR amplifications were performed at least twice with total RNA preparations obtained from a minimum of two independent extractions. **(B)** Promoter sequences of *A. hydrophila* ATCC7966^T^ AHA_1089 and AH-3 *flgT* determined *in silico*. Italic letters indicates Shine-Dalgarno sequences upstream of AHA_1089 and AH-3 *flgT* start codon ATG (gray box). The -12 and -24 show sequences for the σ^54^ binding.

Although the *Aeromonas flgT* is located outside the polar and lateral flagella chromosomal regions it is involved in flagella motility, therefore we analyze whether *flgT* is under the control of some flagella regulator. By RT-PCR, we analyzed the *flgT* transcription in the wild-type AH-3; the non-polar flagella mutants AH-3::*flrA*, AH-3::*flrBC*, and AH-3::*fliA*_p_; and the non-lateral flagella mutants AH-3::*lafK* and AH-3::*lafS*. Data show *flgT* is not transcribed in AH.3::*flrA* and AH-3::*flrBC*, mutants, being transcribed in AH-3::*fliA*_p_, AH-3::*lafK*, and AH-3*::lafS* mutants (**Figure 3**). Therefore, *Aeromonas flgT* is transcribed from a polar-flagellum class III promoter. Furthermore, *in silico* analysis of DNA sequences upstream of AHA_1089 and AH-3 *flgT* show putative σ^54^ promoter sequences (**Figure 3**).

Complementation assays of AH-3Δ*flgT* with pLA-FLGT cosmid or pBAD33-FLGT plasmid induced with 0.2% L-arabinose showed that transconjugants are able to swim in liquid media and have a radial expansion in semi-solid plates identical to that of the wild-type AH-3 (**Figure 2**).

### Role of FlgT in Polar and Lateral Motility

In order to analyze whether *flgT* is also involved in lateral flagella motility we performed two double mutants: a non-polar flagellated and FlgT mutants (AH-3::*flaA*Δ*flaBflgT*) and a non-lateral flagellated and FlgT mutants (AH-3Δ*lafA*Δ*flgT*). Both double mutants are unable to swim in liquid media but whereas motility in soft agar was abolished in the AH-3Δ*lafA*Δ*flgT* mutant, the AH-3::*flaA*Δ*flaBflgT* show a highly reduction in relation to observed in the wild-type AH-3 and similar to AH-3Δ*flgT* and AH-3::*flaA*Δ*flaB* mutants (**Figure 2**). These data suggest that FlgT is only involved in polar flagellum motility and do not affect lateral flagella. Furthermore, AH-3::*flrA* mutant having mutated the polar flagellum master regulator, is unable to transcribe the polar flagellum genes, as well as *flgT* and shows identical motility phenotype as AH-3::*flaA*Δ*flaB* and AH-3::*flaA*Δ*flaBflgT*.

**FIGURE 4:**
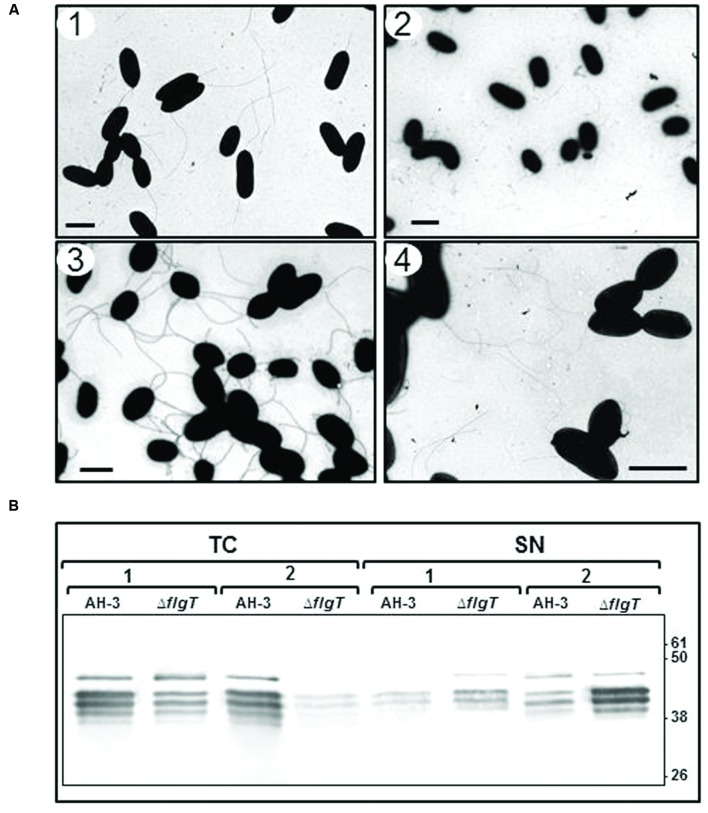
**(A)** Transmission electron microscopy of AH-3Δ*flgT* mutant during the mid-log-phase (OD_600_ ≈ 0.5) (1) and the late-log-phase (OD_600_ ≈ 2) (2) growth at 25°C on liquid media and at the late-log-phase growth in soft agar plates (3). *A. hydrophila* AH-3 during the late-log-phase growth at 25°C on liquid media (4). Bacteria were gently placed onto Formvar-coated copper grids and negatively stained using 2% uranyl acetate. Bar = 2 μm. **(B)** Western-blot of total bacterial cells (TC) and supernatants (SN) of *A. hydrophila* AH-3 and AH-3Δ*flgT* mutant during the mid-log-phase (1) and the late-log-phase (2) growth at 25°C on liquid media, using specific antiserum against purified polar flagellins.

TEM of AH-3Δ*flgT* mutant, grown overnight at 25°C in liquid medium, showed many broken polar flagella not assembled on the bacterial surface. However, grown in soft agar showed the lateral flagella assembled on it (**Figure 4**). Using TEM and western-blot assays, we assessed whether the AH-3Δ*flgT* mutant has a defect in polar flagellum assembly or anchorage. We analyzed in 100 cells of the wild-type AH-3 and the *flgT* mutant, by TEM, the proportion of polar flagellated bacteria at different times of bacterial growth. In the wild-type, AH-3, the number of polar flagellated cells increase over time into the population; however, the number of polar flagellated cells shown a strong reduction in the *flgT* mutant over time. Thus, while in the mid-log phase growth (OD_600_ ≈ 0.5) the 58% of *flgT* mutant population shows an anchored polar flagellum, in the late-log phase growth (OD_600_ ≈ 2) the proportion of polar flagellated cells decreased to 12% (**Figure 4**). Furthermore, to quantify the amount of attached and unattached polar flagellum during growth, we analyzed whole-cells and supernatants of the wild-type AH-3 and the *flgT* mutant in the mid- and late-log phase growth, by western-blot using specific antiserum against purified polar flagellins (Gavín et al., 2002). These assays showed that most polar flagellins are detected in whole-cells of wild-type, because polar flagellum is anchored in the bacterial surface and only a small amount is released in the supernatant, both in mid- and late-log phase growth (**Figure 4**). However, in the *flgT* mutant, the amount of polar flagellins in supernatant, increase during bacterial growth, since the amount of not anchored polar flagellum increases, being higher in the late-log phase than in the mid-log phase (**Figure 4**). Complementation of AH-3Δ*flgT* mutant with pBAD33-FLGT plasmid, under induced conditions (0.2% L-arabinose), restore the anchorage of polar flagellum in the late-log phase growth and reduce the amount of polar flagellum in the supernatant. These data suggest that the reduced number of flagellated bacteria in the *flgT* mutant population was due to a defect in their ability to anchor the polar flagellum to surface.

### Location of FlgT in the Polar Flagella

**FIGURE 5:**
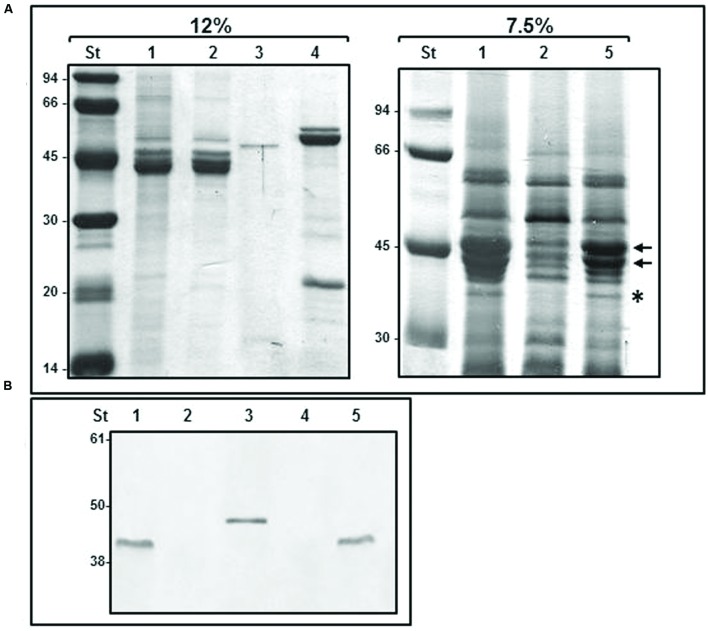
**Purified HBBs of polar and lateral flagella. (A)** 12 and 7.5% SDS-PAGE of purified flagella HBB. The black arrows show FlaA and FlaB polar flagellins. The asterisk shows a band which molecular weight correlate to MotY and MotX proteins. **(B)** Western blot analysis using *A. hydrophila* AH-3 FlgT antiserum (1:1,000). Size standard (St); polar flagella HBB of AH-3 (1), AH-3Δ*flgT* mutant (2); purified His6-FlgT protein (3); lateral flagella HBB of AH-3::*flhA* (4); and AH-3Δ*flgT* mutant complemented with pBAD33-FLGT grown under inducer condition (5).

The evidences that FlgT plays a role in the anchoring of *Aeromonas* polar flagellum to the cell surface prompted us to search its location. In *Vibrio* spp. the orthologs protein has been detected in the periplasmic space and constitutes the H-ring, which is associated with the polar flagellum basal-body (Terashima et al., 2010). In order to locate the *Aeromonas* FlgT, we purified polar flagellum HBB of *A. hydrophila* AH-3 and AH-3Δ*flgT* mutant growth in liquid media at 25°C and analyzed them by SDS-PAGE and Coomasie-blue stained. In a 12% SDS-PAGE, the bands profile of the wild-type and the mutant were similar; however, in a 7.5% SDS-PAGE they showed some differences. The wild-type shows two intense bands around 40 KDa, which correlate with the molecular weight of polar flagellins (FlaA and FlaB) present in the HBBs fraction as a result of the resistance to despolymerization that have the highly glycosylated polar flagellum of *Aeromonas* AH-3. These two bands are strongly reduced in the AH-3Δ*flgT* and also present in the mutant complemented with pBAD33-FLGT grown under inducer conditions (**Figure 5**). Furthermore, the 7.5% SDS-PAGE showed some bands which are absent in the AH-3Δ*flgT* mutant, being one of them correlated with the molecular weight of MotY and MotX proteins that constitute the T-ring of the flagellum basal body. In order to known if one of these absent band correspond to FlgT, we make a transductional fusion of AH-3 FlgT with six histidine residues by cloning the *A. hydrophila* AH-3 *flgT* in the pET-30 Xa/LIC vector. The His_6_-FlgT was overexpressed in *E. coli* and purified protein was used to obtain specific *A. hydrophila* AH-3 FlgT antiserum. Polar flagellum HBBs of *A. hydrophila* AH-3 and AH-3Δ*flgT* mutant were analyzed by western-blot assays using specific *A. hydrophila* AH-3 FlgT antiserum. We only found positive reaction with the purified His_6_-FlgT and with a band of 42 KDa present in the polar flagellum HBB of *A. hydrophila* AH-3 (**Figure 5**). We also obtained the lateral flagella HBB of AH-3::*flhA* mutant, which do not have the FlhA protein of the polar flagellum export-apparatus and is unable to constitute the polar flagella basal body. Western-blot assays using AH-3 FlgT antiserum do not had positive reaction with the lateral HBB (**Figure 5**). Data suggest that FlgT is a component of the polar HBB of *Aeromonas* as previously described in *Vibrio* ssp.

**FIGURE 6:**
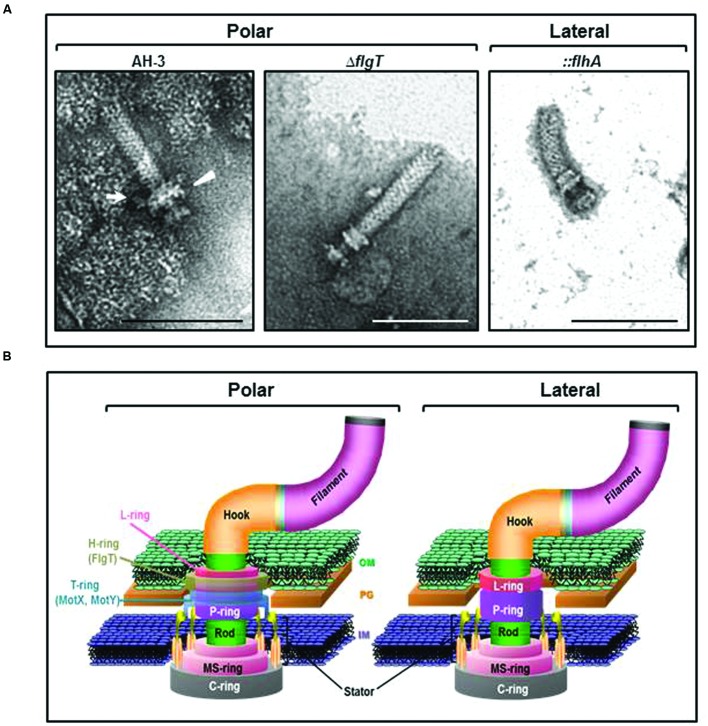
**(A)** Transmission electron microscopy of *A. hydrophila* HBB. Polar flagellum HBB of AH-3 (AH-3) and AH-3Δ*flgT* (Δ*flgT*); and lateral flagella HBB of AH-3::*flhA* (*::flhA*). White arrow points the H-ring and white triangle points the T-ring. The HBB were gently placed onto Formvar-coated copper grids and negatively stained using 2% uranyl molibdate. Bar = 100 nm. **(B)** Diagram of the polar and lateral flagellar basal body of *A.* AH-3. H- and T-rings surround the LP-ring in the polar flagellum basal body of AH-3. OM, outer membrane; PG, peptidoglycan layer; and IM, inner membrane.

To investigate if FlgT constitute a ring around the LP-ring, we performed TEM of purified polar flagella HBBs from AH-3 and the AH-3Δ*flgT* mutant. The HBBs of the wild-type AH-3 have a LP-ring with a protuberance which is not present in the LP-ring of the AH-3Δ*flgT* mutant. Furthermore, the HBBs of AH-3Δ*flgT* mutant also lost the T-ring, consisting for the MotX and MotY proteins. The lateral flagella HBBs of the wild-type AH-3 were structurally similar to the polar HBBs of the AH-3Δ*flgT* mutant (**Figure 6**).

### Adhesion to HEp-2 Cells and Biofilm Formation

**FIGURE 7:**
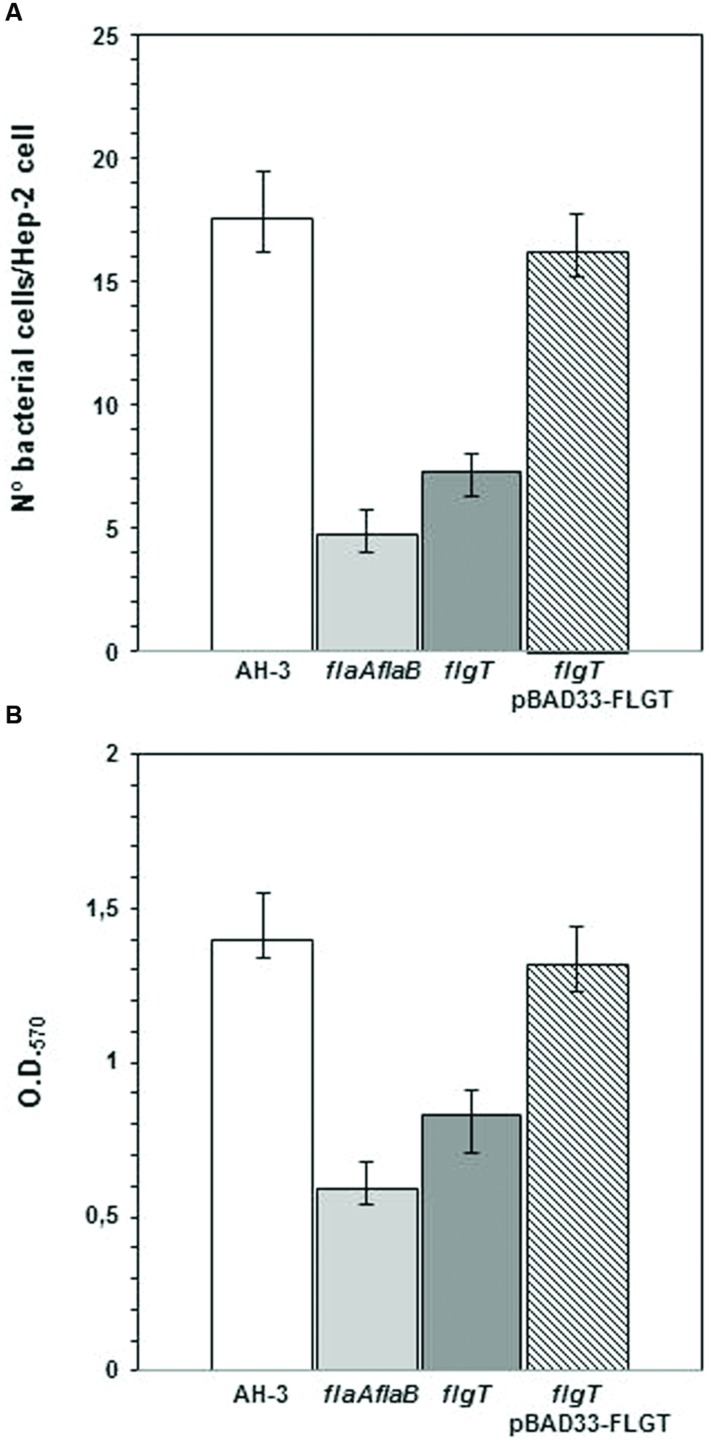
**(A)** Adhesion of *A. hydrophila* AH-3, non-polar flagellum mutant AH-3::*flaAΔflaB*, AH-3Δ*flgT* mutant, and AH-3Δ*flgT* complemented with pBAD33-FLGT to HEp-2 cells. Mean number of adherent bacteria per HEp-2 cells. **(B)** Biofilm formation ability of *A. hydrophila* AH-3, non-polar flagellum mutant AH-3::*flaAΔflaB*, AH-3Δ*flgT* mutant, and AH-3Δ*flgT* complemented with pBAD33-FLGT. OD_570_ quantifies the amount of crystal violet retained by the biofilm on the microtiter plates after staining. The complemented mutant was grown under induced conditions (0.2% L-arabinose). The average of three independent experiments (each experiment performed in duplicate) is show. Error bars represent standard deviations.

In order to correlate polar flagella stability and motility with adherence to mammalian cells, we examined the interaction of *flgT* mutant with cultured monolayers of HEp-2 cells. Differences in adherence were calculated by determining the average number of bacteria adhering to HEp-2 cells (**Figure 7**). We also compared the ability of the wild type and the *flgT* mutant to form biofilms in microtiter plates (**Figure 7**). The *A. hydrophila* wild type strain, AH-3, exhibited an adhesion value of 17.6 (17.6 ± 1.9) bacteria adhered per HEp-2 cell and a biofilm formation ability with an OD_570_ value of 1.43 (1.43 ± 0.15). The mutant lacking FlgT showed a 58.5% reduction in HEp-2 cell adhesion, which is slightly higher than that determined in the non-polar flagellated mutant AH-3::*flaA*Δ*flaB* (72%). The results obtained in biofilm formation (**Figure 7**) show a similar overall pattern to the adhesion, when comparing the characteristics of wild-type and mutant strains. The effects observed in biofilms formation are less marked. Mutants lacking FlgT showed a 40.7% reduction and the non-polar flagellated AH-3::*flaA*Δ*flaB* have a 57.8% reduction (**Figure 7**). Both, adhesion to HEp2-cells and biofilm formation were fully rescued in the *flgT* mutants by the introduction of the wild-type gene.

## Discussion

Mesophilic *Aeromonas* possess a constitutive glycosylated polar flagellum energized by an electrochemical potential of sodium ions. In previous study we described that polar flagellum stator complex is composed of two redundant pairs of membrane proteins: PomAB and PomA_2_B_2_, with different sensitivity to sodium concentrations; and two essential motility proteins (MotXY) which make up the T-ring (Wilhelms et al., 2009; Molero et al., 2011). The analysis of *A. hydrophila* ATCC7966^T^, *A. salmonicida* subsp. *salmonicida* A449, *A. veronii* B565, and *A. caviae* Ae398 genome sequences (Seshadri et al., 2006; Reith et al., 2008; Beatson et al., 2011; Li et al., 2011) revealed an open reading frame which deduced amino acid sequences exhibit 27–28% identity, 46–48% similarity to *Vibrio* spp. FlgT. Hybridization assays using an AHA_1089 DNA probe led to identify an homologous gene in *A. hydrophila* AH-3. As in *Vibrio* spp., upstream of *Aeromonas flgT* we found two open reading frames which encode amino acid sequences orthologs to *flgO* and *flgP;* however, the chromosomal location is different in *Vibrio* spp, *Shewanella oneidensis*, and *A. hydrophila*. In *Aeromonas* these genes are outside the polar flagella chromosomal regions and *flgT* transcribed under the control of a σ^54^ promoter FlrC-dependent, as determined by RT-PCR analysis in polar flagella transcriptional regulators mutants (AH.3::*flrA*, AH-3::*flrBC*, and AH-3::*fliA*_p_). Lateral flagella regulators as LafK and LafS do not control *flgT* transcription (**Figure 3**). As described in *Vibrio* (Terashima et al., 2010), the *Aeromonas* FlgT shows an N-terminal signal peptide for secretion with a cleavage site between Ala^18^ and Glu^19^, which suggest is translocated to the periplasmic space like MotX and MotY, and two conserved cysteine residues that might form a disulfide bond for protein stabilization (**Figure 1**). By constructing specific *flgT* mutants in the wild-type (AH-3Δ*flgT*), a non-polar flagella mutant (AH-3::*flaA*Δ*flaBflgT*) and a non-lateral flagella mutant (AH-3Δ*lafA*Δ*flgT*) we demonstrated that *Aeromonas* FlgT is only involved in polar flagella motility. Single and double mutants are unable to swim in liquid medium; however, motility in soft-agar plates was only abolished in the double mutant unable to form lateral flagella and FlgT (AH-3Δ*lafA*Δ*flgT*). The double mutant unable to produce polar flagella and FlgT (AH-3::*flaA*Δ*flaBflgT*), as well as the single mutants for polar flagella (AH-3::*flaA*Δ*flaB*) or FlgT (AH-3Δ*flgT*) only show reduction of their radial expansion in soft-agar plates, since lateral flagella are able to rotate (**Figure 2**). The swimming phenotype of wild-type was restored when mutants were complemented using the pLA-FLGT cosmid or pBAD33-FLGT plasmid in presence of L-arabinose.

In order to known whether inability to swim was produced by an unassembled polar flagellum or a flagellum unable to rotate, the AH-3Δ*flgT* was analyzed by TEM after grown overnight at 25°C in liquid media. The *flgT* mutant shows many broken polar flagella not assembled on the bacterial surface (**Figure 4**). Analysis of attached and unattached polar flagellum at different times of bacterial growth show that the amount of unattached flagella increases over the phase growth, as reported in *Vibrio cholerae flgT* mutant (Martinez et al., 2010). Thus, in the mid-log phase, more than half of bacterial cells (58%) show attached the polar flagellum, and the amount of polar flagellins is similar in whole cells and supernatant. Nevertheless, in the late-log phase, only a reduced number of cells (12%) show polar flagella attached in its surface, being mostly aflagellate or with broken flagella and the amount of polar flagellins in the supernatant were strongly higher than in whole cells (**Figure 4**). Although the polar flagellum was assembled in the mid log-phase and probably rotates, their rotation in absence of FlgT makes the flagella structure to be unstable and break. Therefore, the more rotate, more unstable is the flagellum structure and the number of aflageladas cells increase in the late log-phase. These results suggest that Aeromonas *flgT* mutant is able to assemble the polar flagellum but probably, it is instable, being its rotation responsible of disbanding from the cell surface. Furthermore, the abolishment of FlgT not affect transcription of class IV polar flagella genes, as was reported in *Vibrio* spp. (Martinez et al., 2010), since the two *Aeromonas* polar flagellines, FlaA and FlaB, which are transcribed from class IV promoters, have detected by specific antiserum in the AH-3Δ *flgT* mutant. Differences in lateral flagella assembly were not detected in the wild-type, AH-3, and the *Aeromonas flgT* mutant after grown in soft-agar plates (**Figure 4**).

Evidences that FlgT plays a role in the stability and anchoring of *Aeromonas* polar flagellum to the cell surface and that *Vibrio* spp. orthologs protein has been associated to the polar flagellum LP-ring (Terashima et al., 2010), in the periplasmic space, prompted us to search the location of FlgT in *Aeromonas*. Purified polar HBB of the wild-type and the *flgT* mutant were analyzed by SDS-PAGE stained with Coomasie-blue and by western-blot, using specific *A. hydrophila* AH-3 FlgT antiserum. The bands profile of the wild-type and the mutant was similar in a 12% SDS-PAGE, however, some differences were visualized in a 7.5% SDS-PAGE (**Figure 5**). The HBBs fraction of wild-type shows two intense bands (around 40 KDa) whose molecular weight correlates with those of polar flagellins (FlaA and FlaB). The high presences of flagellins are a result of the resistance to despolymerization that have the glycosylated polar flagellum of *Aeromonas* AH-3. These two bands are strongly reduced in the AH-3Δ*flgT*because HBB were purified after overnight grown and most polar flagella are released to the supernatant in the mutant. Furthermore, some bands around 32 KDa are absent in the AH-3Δ*flgT* mutant, which correlated with the molecular weight of MotY and MotX proteins that constitute the T-ring of the polar flagellum HBB (Molero et al., 2011). Western-blot assays with specific anti FlgT antiserum shows the presence of FlgT in the polar HBB of wild-type, but absent in the *flgT* mutant. FlgT was not detected in *A. hydrophila* lateral flagella HBBs (**Figure 5**).

Analysis of HBBs by TEM showed polar flagellum HBBs of the *flgT* mutant were similar to lateral flagella HBBs of the *flhA* mutant (polar aflagellated mutant) and did not show protuberances associated to the LP-ring, corresponding to the H- and T-rings (**Figure 6**). As described in *Vibrio* spp (Terashima et al., 2006, 2010), the data suggest that *Aeromonas* FlgT constitute the H-ring associated to the LP-ring and probably anchor the T-ting, whose components are MotX and MotY (Molero et al., 2011). However, in contrast to described in *Vibrio* spp. (Martinez et al., 2010) the loss of the T-ring is not produced by the non-transcription of polar flagellum class IV genes in the *flgT* mutant, but rather probably for its inability to anchor or stabilize the T-ring in absence of H-ring. The absence of T-ring could correlate with the loss of ≈32 KDa bands in the HBB of *flgT* mutant analyzed in 7.5% SDS-PAGE, which may correspond to the lost MotX and MotY (**Figures 5** and **6**)

In our previous research we described that adhesion and biofilms formation of *Aeromonas* is affected for the loss of polar flagellum, as well as for its inability to rotate, since bacterial do not make sufficient contact with the epithelial cells (Canals et al., 2006b). The loss of FlgT reduces progressively during the grown the amount of bacterial cells with an anchored polar flagellum and therefore, the number of motile bacteria. This phenotype leads to a strong reduction of adherence ability and biofilm formation in relation to wild-type, which is somewhat higher than the quantified in a non-polar flagella mutant (**Figure 7**).

Then, our data in *A. hydrophila* suggests that FlgT is present in the HBB of the unsheathed polar flagellum, which is sodium-driven by two different stator complexes. This protein constitutes a substructure in the polar HBB, the H-ring, associated to the LP-ring and it is probably essential for anchorage and stability of the T-ring but is not involved in the transcription of polar flagella genes. Therefore, FlgT is essential for polar flagellum stability and rotation. Furthermore, FlgT is not present in HBB of lateral flagella.

## Author Contributions

SM and JT conceived the study and analyzed the data. SM drafted the manuscript and JT critically commented and revised the manuscript.

## Conflict of Interest Statement

The authors declare that the research was conducted in the absence of any commercial or financial relationships that could be construed as a potential conflict of interest.
